# Medical and Physical Intelligence

**Published:** 1821-08

**Authors:** 


### Medical and Physical Intelligence.

*Copy of the Report to the Secretary of State for the Home Department, from the National Vaccine Establishment; dated* 12*th April,* 1821.

To the Right Honourable Lord Viscount Sidmouth, Principal Secretary of State for the Home Department, &c. &c. &c.

*National Vaccine Establishment, Percy-street;* 12*th April,* 1821.

MY LORD,

IT is with great regret we announce to your Lordship, that the small-pox has occasioned the loss of many lives in various parts of the United Kingdom since our last Report; and that not less than 792 persons have died of that distemper within the Bills of Mortality, in the course of the last year. This is about one-third of the average number of those who perished annually in the metropolis before the introduction of vaccination; but so many deaths afford a strong presumptive proof that great prejudices still prevail against vaccination, and that the benevolent designs of the government are still far from being accomplished.

This Board has laboured incessantly to set forth the comparative ease and safety of the indisposition of vaccination, and the difficulty and danger of the small-pox, whether occurring naturally or occasioned by inoculation; and it has been assisted most importantly, and in the true spirit of disinterested benevolence, by the Master, Governors, and Members of the Court of Assistants, of the Royal College of Surgeons, who have bound themselves individually to each other, by a solemn engagement, not to yield to any solicitations to inoculate for the small-pox. This good example has been followed by most of the respectable practitioners in the country; though some of them, we are sorry to say, have lent themselves improvidently to this injurious practice. And we find that the multitude in many places has been so infatuated as to accept the proffered services even of itinerant inoculators, in spite of their gross ignorance of all disease, and of the rudeness and inaptitude of the instruments which they employ to insert the poison. Hence a perpetual source of contagion is supplied and kept up, to the constant danger of all such as have not yet been vaccinated, or who may have undergone an imperfect process, or whose peculiarity of constitution makes them still susceptible of the variolous disease; a peculiarity similar to that which renders some persons capable of taking the small-pox twice; of which, within the period of three years only, we have received evidence of not less than fifty-two instances.

It is true, indeed, my Lord, that we have received accounts from different parts of the country, of numerous cases of small-pox having occurred after vaccination; and we cannot doubt that the prejudices of the people against this preventive expedient are assignable (and not altogether unreasonably, perhaps,) to this cause.

These cases the Board has been industriously employed in investigating; and, though it appears that many of them rest only on hear-say evidence, and that others seem to have undergone the vaccine process imperfectly, some years since, when it was less well understood and practised less skilfully than it ought to be, yet, after every reasonable deduction, we are compelled to allow that too many still remain on undeniable proof, to leave any doubt that the pretensions of vaccination to the merit of a perfect and exclusive security in all cases against small-pox, were admitted at first rather too unreservedly. Yet the value of this important resource is not disparaged in our judgment; for, after all, these cases bear a very small proportion to the number of those who are effectually protected by it. The reports of the vaccinators at the several stations in the metropolis give only eight cases of small-pox, out of nearly 67,000 vaccinated by them, since the first establishment of this Board; and, as the small-pox has prevailed extensively in London, these persons so vaccinated must have been frequently exposed to contagion, and consequently the protecting effect of vaccination must have been submitted to as severe a test as can well be imagined. Moreover, we have the most undoubted proofs, from experience, that, where vaccination has been performed perfectly, small-pox occurring after it, is almost universally a safe disease; and, though ushered in by severe symptoms, has hardly ever failed to be cut short before it had reached that period at which it becomes dangerous to life.

This controlling power of vaccination must be admitted as next in importance to its preventive influence; and surely justifies our high estimation of the value of this great discovery.

The Board has taken care to promulgate to all its correspondents, by repeated admonitions, those modes of conducting the process of vaccination, which ample experience, within its own immediate sphere of observation, has ascertained to be the most successful.

It cannot be denied, my Lord, that the continuance of the practice of inoculation for the small-pox is the main source of whatever disappointment we have met with; for, in those countries where the legislature has interfered to prohibit it, and to enforce vaccination, the small-pox has become almost unknown, and the full benefit of this valuable discovery is enjoyed; but similar results, we know, cannot be looked for in the United Kingdom, until the whole community shall concur voluntarily in its salutary practice.

We have only to add, that 6,933 persons were vaccinated last year at the several stations in London; that 48,105 charges have been given to the public; and that 77,467 have been vaccinated in Great Britain and Ireland, by our immediate correspondents only; making a total of 84,400 vaccinated last year, a number superior to that of any former year.

HENRY HALFORD, *President.*

RICHARD POWELL, M.D.

JOHN COOKE, M.D.

WILLIAM MACMICHAEL, M.D.

P. MERE LATHAM, M.D.

} *Censors of the Royal College of Physicians.*

T. FORSTER, *Master of the Royal College of Surgeons.*

EVERARD HOME,

WILLIAM BLIZARD,

} *Governors of the Royal College of Surgeons.*

By order of the Board,
JAMES HERVEY, M.D. *Registrar.*

---

*To the Editor of the Medical and Physical Journal.*

THE possibility of a slight hint sometimes exciting others to grave researches, and leading to some useful discovery, which we have not leisure or ability to institute or produce ourselves, has caused me to address you in the present instance.

Some weeks ago, I read an account (for the truth of which I can, of course, not vouch,) that the king's stag-hounds had been sent to Brighton for the benefit of sea-bathing, by the order of the master of the hounds; and that this order was given because symptoms of madness had shown themselves among them. I was much astonished to find that, in our days, any reliance could yet be placed, even by a layman in the healing art, on such a remedy as sea-bathing, in such a disease as hydrophobia; and the wish that some more rational method of treatment than we at present possess might at last be propagated, was strongly raised in me. This brought to my recollection, that the professor, whose veterinary lectures I attended on the continent, once stated that the probable cause of the *rabies canina* was the disparity of the number of female dogs who were suffered to live to the males, and the consequent impossibility with many of the latter of fulfilling the call of nature for sexual intercourse. As some proof for the truth of this position, he further stated that all the individuals to whom he could ever trace the origin of hydrophobia had been invariably male dogs. As I do not remember having heard this circumstance mentioned since I have been in this country, I am desirous of submitting an account of it to your numerous readers, in the hope that, if it is really new to them, some one might be instigated to further inquiry.

K. LANG.

*Kensington; July* 14*th*, 1821.

---

THE following observations and hints have been sent to us by a physician of the University of Edinburgh, who, very properly, thinks it is not necessary that his name should be attached to them in order that they may be inserted in this Journal.

"It is asserted by Bajon,* that the virus of yaws is capable of being communicated to domestic animals; and, when it appears among the fowls, the disease spreads so rapidly that, to check it, those affected with the complaint must be immediately killed. Dogs are equally liable to be affected, and in these animals it assumes very much the appearance of the venereal disease."†

No plausible account of the origin of the venereal disease has yet been published, although much has been said upon it by different writers. The above quotation from a note contained in Winterbottom's Account of Sierra Leone, suggests the notion that the venereal disease may be a variety of *frambœsia*, or the yaws. That contagious diseases are capable of being communicated from one species of animals to other species, is proved by the facts which we know of hydrophobia and vaccinia; and, it is probable, many other instances of a similar kind would be observed, if we were better acquainted with the diseases of brutes. The preceding quotation supplies an example of the communication of a contagious disease, the yaws, from man to fowls and dogs; in which last species of animals it is said to assume very much the appearance of the venereal disease. Now, may not frambæsia be so modified and altered in the constitution of the dog, as to present certain new qualities and symptoms, which it did not possess as it originally existed in the human constitution? And may not the form under which the disease presents itself in the dog be really the venereal disease, or, at least, the source whence that disease in man is derived? These suppositions acquire some degree of probability from the evidence of Bajon; nor do the solitary experiments of John Hunter, wherein he failed to communicate syphilis to a dog, appear to detract much from their credulity; but the facts would require to be well authenticated before any positive inference could be drawn from them.

It should be recommended to physicians who reside in countries where frambæsia is epidemic, to perform experiments with the virus of that disease upon different animals, so as to ascertain with precision the relative nature of syphilis and frambæsia. It is only by attentive observation and accurate experiment that discoveries can be made; and no suggestion is entirely without use, which, by exciting the ardour of inquiry, leads to more correct information on any important subject."

---

Dr. Cagnola, of Milan, proposes (*Annali Universali di Medicina*, Nov. 1820,) the prussic acid as a means of killing the whole of a *tænia cucurbitina*, in the case of part of it appearing without the anus. Authors heretofore only recommended that it should be pulled

---

* *Histoire de Cayenne.*

† *An Account of the Native Africans in the Neighbourhood of Sierra Leone.* By Thomas Winterbottom, M.D. Vol. ii. p. 153.

at in various ways, in order that the whole may be withdrawn; but this practice is hardly ever successful, the worm generally breaking when any considerable force is used. Dr. Cagnola thinks that, if the part without the anus were touched by the prussic acid prepared according to Gay-Lussac's process, the whole of the worm would be instantly deprived of life. It is hardly necessary to remark, that great caution must be used if such a measure be employed; especially that the return of the portion of worm touched by the prussic acid into the intestines may be securely guarded against.

---

*Action of Cork on Chalybeate Waters.*—Mr. Wurza, on examining some bottles of chalybeate waters, was surprised to find no signs of iron in them; and, on seeking the cause of this circumstance, he discovered it in the astringent nature of the corks, which had combined with the metallic substance. He advises, when chalybeate waters are kept in bottles, that the corks should be first well steeped in the waters, in order that the astringent matter they contain may be saturated with the iron.

---

In the account given of the proceedings of the School at Alfort, for the improvement of Agriculture and Veterinary Medicine, we find the following observations especially interesting to medical men.

An accumulation of serous fluid into the cavity of the pleura is very generally observed in animals which have died from poisoning by arsenic: in order to ascertain whether or not this appeared to be owing to absorption of any part of the mineral, the mediastinum of a horse, poisoned by the white oxide of arsenic, was submitted to chemical analysis by Mr. Lassaigne; when the presence of the arsenic in these membranes was proved.

---

*Account of a Chinese Lusus Naturæ.* By John Livingstone, Surgeon to the British Factory, China.

A-ke was born sixteen years ago, in the district of Yun-lang-yuen, with another male child of nearly the same size united to the pit of his stomach by the neck, as if his brother had plunged its head into his breast. The skin of the principal here joins that of the upper part of the neck of the parasite, quite regularly and smoothly, excepting the superficial blood-vessels, which appear somewhat turgid. The sufferings of the mother were so great, that she survived the birth of this monster only two days.

Since that time the parasite has not much increased in size,* and at

---

* I have the authority of Lieut-General Wood for stating, that a careful admeasurement of the parasite was made at his request:—the trunk and neck measured about eleven inches, and the longest limb thirteen inches, making the extreme length two feet. This accords sufficiently well with the size I have mentioned; but, as the modellers in China do not work by any scale, it would be useless to deduce any *exact* measurement of the whole figure by knowing a part.

present is not much larger than new-born infants usually are; but the bones are completely formed. The shoulder-bones are remarkably prominent. But all this plumpness has disappeared from the original, whose bones seem only to be covered with skin.

The attachment of the neck of the parasite to the chest of the principal, admits of a semirotatory motion. The natural position of the bellies is towards each other; but A-ke can turn his brother so far round that he can bring either side towards his own belly. He also shows that his brother's arms can be moved freely. The thighs and legs remain stiffly bent, as represented in the model: the thigh being anchylosed with the ossa innominata above, and the tibiæ below. In sciagraphia, genitalia nimis perfecta apparent; quoniam in archetypo, testium vestigium nullum, scrotique exiguum tantum, videndum sit. At penis proportionaliter crassus est; et præputium glandem semivelat. Tentigo interdum observatur; quo tempore fluidum viscidum ex urethra stillat, quapropter Sinenses semen copiose secerni credunt. Renes officia rite perficiunt; anus deest.

A-ke is now about four feet ten inches high, of a feeble frame and sickly appearance; but, excepting the encumbrance above described, he is in all respects perfectly formed. He appears to be sufficiently conversible and intelligent, and says that he has the same feeling of pain, if any part of his brother's body is hurt, as if it was the same part of his own body: even the slightest touch which would be perceptible if applied to his own person, is equally perceptible if applied to his brother. This statement was most satisfactorily confirmed by an ingenious medical gentleman; who, observing A-ke's attention to be fully employed, and his head turned away in a contrary direction, pinched quickly the hip of the parasite: A-ke instantly struck the same part of his own person, just as if that had been the pinched place.

Formerly he had reason to imagine, from certain obscure motions which he perceived within his brother when he was himself in pain, that all their feelings were reciprocal; but for some time past he has not been sensible of this, nisi micturus sit. Frater ejus nunquam eodem tempore, seu urgente naturâ, seu curiositati adstantium satisfaciendi causa, urinam reddere deficit.

A-ke's respiration is never perfectly free: on the contrary, it is commonly laborious; and, on the slightest exertion, such as walking to a little distance, ascending a flight of steps, or the like, he breathes quickly, and with difficulty. To relieve this, he supports the parasite with his hands; but, to obtain any considerable degree of ease, a recumbent posture is necessary. His pulse is commonly quick and small. Mr. Gomez felt distinctly the pulsation of the carotids in the neck of the parasite; it was feeble. He also examined carefully the pulse at the wrists; it was very slow, (*valde lente.*)

The usual temperature of both is natural. A-ke wears an unusual quantity of clothes, yet he never appears to perspire, even in the warmest weather. His usual gait is unsteady and feeble: when he walks up or down stairs, he supports himself with one hand, and his brother with the other, and brings both his feet upon the same step before he attempts to advance another foot.

When in his best state of health, he informed Mr. Gomez his appetite was so good that he could take as much food as any three of his age; at present, his health in general is much impaired. He complains of weakness of stomach, loss of appetite, defective and painful digestions; so it is commonly thought that he cannot live long.

---

*Extract from* CAPTAIN PARRY'S *Journal of a Voyage of Discovery to the Arctic Regions.*

THE greatest cold experienced by Captain Parry was quite tolerable in calm weather. One of the crew of the Griper, who had lost his way in a hunting excursion, returned with one of his hands much frost-bitten. It was at first as hard as a piece of marble, but, by successful treatment, it recovered so far that he lost only a part of each of the four fingers of his left hand. Another sailor, who had his hands frost-bitten, came on-board in such a state that, when his hands were immersed in a tub of cold water, for the purpose of being thawed, the cold communicated to the water created a film of ice on its surface. The skin and nails came off some of the fingers, and the rest were amputated. One of the most remarkable effects, however, of severe cold, was its influence on the mental as well as the corporeal faculties. On the 5th of October, two of the gentlemen of the expedition, who had exposed themselves to severe frost in the ardour of pursuing a wounded stag, were sent for by Captain Parry. Upon arriving in his cabin,

"They looked wild, spoke thick and indistinctly, and it was impossible to draw from them a rational answer to any of our questions. After being on-board for a short time, the mental faculties appeared gradually to return with the returning circulation; and it was not till then that a looker-on could easily persuade himself that they had not been drinking too freely. To those who have been much accustomed to cold countries, this will be no new remark; but I cannot help thinking that many a man may have been punished for intoxication, who was only suffering from the benumbing effects of frost: for I have more than once seen our people in a state so exactly resembling that of the most stupid intoxication, that I should certainly have charged them with that offence, had I not been quite sure that no possible means were afforded them on Melville Island to procure any thing stronger than snow-water."—*Captain Parry's Journal*, p. 108-9.

The only other affliction which arose from the weather, was what is called in America *snow-blindness*. It began by a sensation like that which is felt when sand or dust gets into the eyes. A solution of sugar-of-lead removed the complaint in two or three days, and the recurrence of the disease was prevented by the use of a piece of crape.

The scurvy appeared in the months of March and April; but the invalids all recovered, in consequence of Captain Parry's having been at much pains to raise some mustard and cress for them in his own cabin.

*Audibility of Sounds.*—Captain Parry was surprised at the great distance at which sounds were heard in the open air, during the continuance of intense cold; and, notwithstanding the frequency with which they had occasion to remark it, it always afforded them surprise. "We have, for instance," says he, "often heard people distinctly conversing, in a common tone of voice, at the distance of a mile; and to-day I heard a man singing to himself as he walked along the beach, at even a greater distance than this."

As Captain Parry does not seem to be aware of the cause of this very curious and important fact, we shall endeavour to explain it, upon principles which Humboldt has ably developed, in a paper on the Increase of Sounds during Night. If the air were at all times of uniform density, the sounds propagated through it would always be of the same intensity; but, in the day-time, the inequality with which different parts of the ground are heated occasions a difference of density in the adjacent air, and thus produces undulations of different density, by which the sonorous undulations are divided and interrupted, in the same manner as light is in passing through alcohol and water imperfectly mixed, or any medium of heterogeneous density. Now, at Winter Harbour, the temperature of the ground and that of the air near it had a wonderful degree of uniformity. The absence of the sun prevented any difference of density arising from the unequal heating of the ground; and, consequently, the mass of air, above a mile long, which, on the 11th February, intervened between Captain Parry and the sailor whose song he heard through that interval, was perfectly homogeneous, and offered no obstruction to the undulations which the voice of the sailor had propagated through it. In proof of this opinion, we have only to look at the temperature of that day, and we shall find that the maximum and minimum temperatures were 38° and 42°, and the mean 39°77; the whole range of temperature for that day being only 4°! which is a satisfactory proof of the perfect homogeneity of the strata of air incumbent on the ground.—(*Edinb. Phil. Journal.*)

---

Dr. Girard, of Lyons, has published a statement, that the liquor ammoniæ, taken in the dose of seven or eight drops, immediately removes the state of drunkenness. Dr. Chantourelle, who was charged to make a report on this subject to the Medical Society of Paris, thought it might be supposed that this effect arose from decomposition of the wine by the ammonia: but some chemical experiments showed that such was not the case.

---

It is known that light is emitted from organized bodies when putrefaction takes place, under certain circumstances: the same phenomenon sometimes occurs in wounds, and doubtless a greater number of instances would be recorded were they often dressed in the dark.

A.

Baron Percy, who, during twenty-five years of wars, has had under his care more than a million wounded, has often been deprived of the advantage of light. It was thus that he observed, in a young soldier, the phosphorescence of a slight wound in the leg for more than fifteen days. In this case it might, perhaps, be attributed to the man's having applied compresses dipped in urine to the wound: but some time afterwards, at the siege of Manheim, a vivid light, a true ignis fatuus, existed for more than six days over the wound of an officer, who had been dressed with compresses wetted with pure water only. Baron Percy has since had frequent opportunities of observing similar facts. —(*Analyse des Travaux de l'Academie des Sciences de Paris, pour* 1819. *Par M. le Baron* Cuvier, *sec. perpet.*)

---

### MONTHLY CATALOGUE OF MEDICAL BOOKS.

### METEOROLOGICAL JOURNAL.

By Messrs. William Harris and Co. 50, Holborn, London.

*From June* 20 *to July* 19, *inclusive.*

| Day of Month. | Moon. | Rain gauge | THERM. | | | BAROM. | | DeLuc's Hygro. | | WIND. | | ATMOSPHERIC VARIATION. | | |
|---|---|---|---|---|---|---|---|---|---|---|---|---|---|---|
| Jun. 20 | | | 54 | 59 | 48 | 30·09 | 30·09 | 55 | 52 | N | N | Cloudy | Fine | |
| 21 | | | 54 | 59 | 47 | 30·08 | 30·14 | 54 | 54 | NE | NNE | Fine | | |
| 22 | ☽ | | 53 | 58 | 52 | 30·19 | 30·20 | 54 | 56 | N | NNW | Fine | Cloud. | Fine |
| 23 | | | 56 | 58 | 49 | 30·19 | 30·19 | 55 | 57 | NE | NNE | Cloudy | | |
| 24 | | | 52 | 57 | 53 | 30·15 | 30·11 | 55 | 57 | NNW | NE | Cloudy | | |
| 25 | | | 59 | 63 | 53 | 30·14 | 30·18 | 55 | 55 | ENE | NE | Cloudy | Fine | Cloud. |
| 26 | | | 58 | 63 | 53 | 30·11 | 30·10 | 56 | 55 | NE | NE | Fine | | |
| 27 | | | 55 | 67 | 55 | 30·11 | 30·15 | 54 | 55 | NE | NE | Fine | | Cloud. |
| 28 | | | 59 | 67 | 54 | 30·15 | 30·16 | 53 | 55 | N | E | Fine | | |
| 29 | ● | | 57 | 67 | 59 | 30·16 | 30·06 | 54 | 53 | ESE | W | Fine | | |
| 30 | | ·26 | 65 | 72 | 61 | 29·99 | 29·80 | 53 | 57 | SW | S | Fine | Rain | |
| July 1 | | ·39 | 63 | 68 | 50 | 29·62 | 29·73 | 58 | 60 | WSW | ESE | Cloudy | | Rain |
| 2 | | ·25 | 52 | 56 | 49 | 29·81 | 29·82 | 58 | 56 | E | E | Rain | Cloud. | |
| 3 | | | 53 | 56 | 45 | 29·82 | 29·96 | 57 | 56 | E | NE | Cloudy | | |
| 4 | | | 54 | 59 | 53 | 30·04 | 30·17 | 55 | 55 | NE | WNW | Fine | | |
| 5 | | | 59 | 64 | 56 | 30·20 | 30·14 | 54 | 55 | N | NW | Fine | | Cloud. |
| 6 | | | 60 | 63 | 53 | 29·95 | 29·83 | 56 | 57 | WNW | W | Cloudy | | Rain |
| 7 | ☾ | | 56 | 59 | 49 | 29·83 | 30·00 | 57 | 57 | NNE | NE | Cloudy | Sho'ry | Cloud. |
| 8 | | ·35 | 55 | 60 | 55 | 30·02 | 30·12 | 57 | 57 | NNE | NE | Fine | | |
| 9 | | | 60 | 66 | 58 | 30·12 | 30·12 | 54 | 54 | NNW | NNW | Fine | | |
| 10 | | | 62 | 62 | 55 | 30·13 | 30·13 | 55 | 56 | NW | NNW | Fine | | |
| 11 | | | 57 | 61 | 54 | 30 16 | 30·13 | 57 | 56 | NE | E | Cloudy | | |
| 12 | | | 58 | 62 | 52 | 30·10 | 30·07 | 55 | 55 | E | SE | Cloudy | Fine | |
| 13 | | | 59 | 63 | 50 | 30·00 | 29·91 | 54 | 55 | SSW | WSW | Fine | Cloud. | |
| 14 | | | 55 | 69 | 55 | 29·83 | 29·81 | 54 | 55 | WSW | WNW | Cloudy | Fine | |
| 15 | ○ | ·26 | 57 | 59 | 55 | 29·71 | 29 82 | 57 | 57 | W | WNW | Rain | | Fine |
| 16 | | | 60 | 65 | 58 | 30·00 | 30·12 | 58 | 57 | N | WNW | Fine | | |
| 17 | | | 64 | 67 | 57 | 30·24 | 30·33 | 56 | 56 | NW | SW | Cloudy | Fine | |
| 18 | | | 64 | 69 | 59 | 30 33 | 30·26 | 54 | 54 | SSE | SE | Fine | | |
| 19 | | | 66 | 71 | 62 | 30·12 | 29·88 | 57 | 57 | ESE | ESE | Fine | | Lightn |

The quantity of rain fallen in the month of June, is 2 inches and 27-100ths.

